# The Homologous Recombination Repair Pathway is Associated with Resistance to Radiotherapy in Nasopharyngeal Carcinoma

**DOI:** 10.7150/ijbs.37302

**Published:** 2020-01-01

**Authors:** Zhihai Wang, Wenqi Zuo, Quan Zeng, Yanshi Li, Tao Lu, Youquan Bu, Guohua Hu

**Affiliations:** 1 Department of Otorhinolaryngology, the First Affiliated Hospital of Chongqing Medical University, Chongqing 400016, China.; 2 Department of Biochemistry and Molecular Biology, Molecular Medicine and Cancer Research Center, Chongqing Medical University, Chongqing, 400016, China.

**Keywords:** Nasopharyngeal carcinoma, radioresistance, homologous recombination repair, NFBD1/MDC1, DNA damage response

## Abstract

Radiotherapy plays a major role in the management of nasopharyngeal carcinoma (NPC). However, the radioresistant cells limit its efficiency and drive recurrence inside the irradiated tumor volume leading to poor outcome for patients. To illuminate the signal pathway involved in the radioresistance and evaluate the potential for predicting NPC response to radiotherapy, we established the radioresistant NPC cell line (CNE2-RR) derived from NPC cell line CNE2 by gradually increased the radiation dose (total 60 Gy), and the radioresistance of CNE2-RR cells was evaluated by the colony formation, FCM and comet assays. Furthermore, comparison of established CNE2-RR cell line to parental cell line found the homologous recombination repair (HRR) proteins differences involved in NPC radioresistance. In addition, the differentially expressed proteins were further validated by western blotting, immunofluorescence and IHC in tumor xenografs and radioresistant NPC tissues. Furthermore, the correlation of HRR proteins expression levels with NPC radioresistance were evaluated. The results showed that the upregulation of HRR proteins were significantly correlated with NPC radioresistance. In addition, using the Youden Index cutoff value, a panel of the HRR proteins analyses revealed a sensitivity of 70%, specificity of 72%. Furthermore, silencing NFBD1 enhanced the radiosensitivity of CNE2-RR cells by impairing IR-inducing γ-H2AX and HR proteins foci formation. These results suggest that controlling the HRR signaling pathway may hold promise to overcome NPC radioresistance.

## Introduction

Nasopharyngeal carcinoma (NPC) is a non-lymphomatous, squamous cell carcinoma that occurs in the epithelial lining of the nasopharynx, which is a prevalent tumor in people of southern Chinese ancestry in southern China and Southeast Asia, and the incidence is still increasing^1^. The disease tends to be more sensitive to ionizing radiation (IR) than other head and neck cancers, therefore, radiotherapy is routinely used to treat patients with NPC. Nevertheless, some of the NPC patients present local recurrences and distant metastases after radiotherapy and the majority of these patients surrender recurrence and metastasis 2, 3. One reason for these failures following radiotherapy is the radioresistance of a subpopulation of NPC clones within the tumor. Radioresistance remains a serious obstacle to successful treatment in these cases. Hence, revealing the molecular mechanism of NPC radioresistance and identifying subgroup of radioresistant NPC patients are urgently needed for personalized therapy.

The efficacy of IR is based on the induction of a broad spectrum of DNA lesions, which in replicating cells often results in deleterious DNA double-strand breaks (DSBs) 4-6. DSBs induced IR are mainly repaired by non-homologous end joining (NHEJ) and homologous recombination (HR) 7, 8. NHEJ mainly participates in DNA damage repair in nonproliferating cells, thus inhibiting NHEJ is putatively toxic to normal cells and may, in general, not provide the desired selectivity for cancer treatment. Whereas, highly replicative cells, such as cancer cells, are more committed to HR-mediated DSBs repair and therefore rely heavily on functional DNA repair pathways for survival 9, 10. Therefore, understanding the pathways involved in cellular DNA repair processes opens a novel rational approach for uncovering the molecular mechanism of NPC radioresistance. Moreover, the recent realization that some cancers have impaired or inactivated HR repair pathway, which can also be explored to design drugs targeting those cancer cells specifically, thereby improving therapeutic outcome and/or potentiating the therapeutic effect of currently used chemotherapeutic drugs 11-15.

In this study, we established radioresistant NPC cell line CNE2-RR to illuminate the mechanism of NPC radioresistance, and found that the HRR pathway related genes NFBD1, BRCA1, BRCA2, RAD51 and RPA1 are involved in the radioresistance of NPC. Furthermore, we validated the potential signaling pathway proteins identified in xenograft tumors and radioresistant NPC tissues, and investigated their predictive values for the response of NPC patients to radiotherapy. In addition, we used lentivirus-mediated shRNA to explore functions of one of identified proteins, NFBD1 (also known as KIAA01770 or MDC1), for radiosensitivity using CNE2-RR cells. Our results indicate the HR signaling pathway is involved in NPC radioresistance and targeting the pathway may hold promise to improve NPC radiosensitivity.

## Materials and Methods

### Patients and Tissue Specimens

This study was approved by Research Ethics Committee of Chongqing Medical University. Written informed consent from all patients or their guardians was obtained. From December 2015 to November 2018, 100 consecutive NPC patients (66 male, 20 female, range, 17-68 years, mean age, 47 years), without distant metastasis at the time of diagnosis, comprising 50 radioresistant and 50 radiosensitive patients, were recruited in this study. All patients accepted a standard regimen of radiotherapy. At the third month after treatment, contrast-enhanced CT/MR scan and thorough examination were performed to evaluate short-term efficacy. According to the RECIST guideline (version 1.1), complete response (CR) and partial response (PR) were divided into radiosensitivity group, while no change (NC) and progressive disease (PD) were divided into radioresistant group 16. The clinicopathologic characteristics of NPC tissues used in the present study are demonstrated in Supplementary Table S1.

### Antibodies and reagents

The lentivirus-mediated shNFBD1 and shControl were purchased from Genechem, Shanghai, China. Hoechst 33342 were purchased from Beyotime Institute of Biotechnology (Nantong, China).The antibodies used in this study were anti-NFBD1 and RPA1 (Abcam, UK); anti-RAD51, anti-BRCA1 and anti-BRCA2 (Santa Cruz Biotechnology, USA); anti-γ-H2AX and anti-phospho-histone H3 (Ser10) (Cell Signaling Technology, Danvers, MA, USA).

### Generation of radioresistant cell lines

Poorly differentiated CNE2 cells were obtained from the Molecular Medicine and Cancer Research Center, Chongqing Medical University, and plated in T25 flask in RMPI-1640 medium (HyClone, Logan City, Utah, USA) with 10% fetal bovine serum (HyClone, Logan City, Utah, USA) at 37 °C with 5% CO_2_, and exposed to various doses from 0 to 10 Gy. The radiation was delivered at room temperature at 300 cGy/min with a linear accelerator (2100EX, Varian, USA). The culture medium was changed after irradiation and returned to the incubator for further incubation. Then, cells were irradiated again when they reached to 80% confluence. Similar procedures were performed with the irradiation frequencies of 2 Gy 2 times, 4 Gy 2 times, 6 Gy 2 times, 8Gy 2 times and 10Gy 2 times to the total dose 60 Gy, to establish the radioresistant cell populations. We defined the fifth generation cells as the radioresistant cell line and named for CNE2-RR. Experiments were performed with the CNE2-RR cells within 4 to 10 passages after the termination of irradiation.

### Colony formation assay

Cells were seeded at low density and irradiated with various doses of IR. Cells were left for 10-14 days at 37 °C with 5% CO_2_ to allow the colonies to form, then fixed with 70% ethanol and stained with 0.5% crystal violet. Colonies containing 50 or more cells were counted as survivors.

### Flow cytometry (FCM)

Cells were seeded onto six-well plates at the density of 10× 10^4^ cells per well and irradiated with various doses of IR. For apoptosis analysis, cells were harvested and stained using Annexin V-FITC Apoptosis Detection kit (Beyotime Institute of Biotechnology, Nantong, China) according to manufacturer's recommendation. For γ-H2AX and G2/M checkpoint analysis, the cells were fixed with ethanol, re-suspended in PBS containing 0.25% (vol/vol) Triton X-100, incubated on ice for 15 min and then incubated in PBS containing 1% BSA and γ-H2AX or phospho-histone H3 (Ser10) antibody for 1 h at room temperature. Samples were then incubated for 30 min at room temperature with Alexa fluor 488 donkey anti-rabbit conjugated secondary antibodies and were determined analyzed by a FACSVantage SE system (BD Biosciences).

### Hoechst 33342 staining

Cells were cultured in 24-well plates for 24 h, exposed to 4Gy, and then fixed with 4% paraformaldehyde for 1 h at room temperature, and stained with Hoechst 33342 in the dark for 30 min. Images were acquired under a Leica MD2700M fluorescence microscope (German).

### Comet assay

Comet assays were performed as described elsewhere 17. The Comet Assay kit (Trevigen Inc., Gaithersburg, MD, USA) was used under alkalic conditions according to the manufacturer's specifications. Comets were visualized using a Leica MD2700M fluorescence microscope (German). The tail moments (TMs) of comets were scored using CASP software.

### RNA extraction and real-time quantitative RT-PCR (qRT-PCR)

Total cellular RNA was extracted using TRIzol reagent (Invitrogen, Carlsbad, CA, USA) and synthesized cDNA using the One-Step SYBR PrimeScriptTM RT-PCR Kit II (TaKaRa Biotechnology, Dalian, China) according to the manufacturer's instructions. The qRT-PCR was performed using SYBR Premix Ex Taq in a LightCycler 480 qRT-PCR system (Bio-Rad; Hercules, CA, USA). The qRT-PCR primers of NFBD1 were NFBD1-F (AGCAACCCCAGTTGTCATTC) and NFBD1-R (TCCACCACCCTGTTGCTGTA). The 2^-∆∆Ct^ method was used to determine the relative quantification of NFBD1 expression.

### Immunofluorescence

Immunofluorescence was performed as described elsewhere 17, 18. Briefly, cells were fixed, permeabilized, washed, blocked, and then primary antibodies were applied overnight at 4 °C, and the appropriate secondary antibodies were applied for 60 min at room temperature. Finally, the cells were washed three times in PBS, and the DNA was stained using DAPI (Sigma-Aldrich, St. Louis, MO, USA) at 5ng/ml. The slides were observed under a Leica MD2700M fluorescence microscope (German).

### Western blotting

Total protein extracts from cells were prepared using RIPA buffer (Beyotime Institute of Biotechnology, Nantong, China), fractionated in SDS-polyacrylamide gels, transferred to polyvinylidene fluoride (Millipore, Billerica, MA, USA), and western blotting were performed by using the appropriate antibody. Antibody/antigen complexes were detected using ECL (Western Bright Sirius; Advansta, Inc., Menlo Park, CA, USA) and images were acquired using an enhanced chemifluorescence detection system (Amersham Biosciences, Piscataway, NJ, USA) under the room temperature.

### Xenograft experiments

All animal husbandry and experiments were performed under a protocol approved by Institutional Animal Care Committee at Chongqing Medical University. CNE2 cells (6.0 × 10^6^) in 0.2 ml of growth medium were subcutaneously injected into the axilla of the Balb/c nude mice. Five animals per group (n=4) were used in control and combination treatment. When the tumor diameter was approximately 4 mm, the treated nude mice were randomized into radiation group and non-radiation group. The radiation group was received 2 Gy of radiation once every other day (total 10Gy) using a linear accelerator (Clinac 2300C/D; Varian, Palo Alto, CA, USA). Tumor size was measured every week and tumor volume =1/2 × length × width^2^. Mice were killed after 35 days after cell injection, and then the tumors were excised, weighed and embedded in paraffin.

### Immunohistochemistry

Standard immunohistochemical procedures were used for xenograf and human NPC tissues using our published method 19, 20. The xenograf tumors were collected five weeks after treatment. At least five 40 × fields were scored. In addition, the five proteins were individually and, as a penal, assessed for its ability to discriminate between radiosensitive and radiaoresistant NPC patients by evaluating its receiver operating characteristic (ROC) curve.

### Statistical analysis

Statistical comparison of mean values was performed using ANOVA, rank sum test (non-parametric statistics) or chi-square (χ^2^) test. Differences with a P-value of < 0.05 were considered statistically significant.

## Results

### Establishment and validation of radioresistant NPC cell line

**Figure 1:**
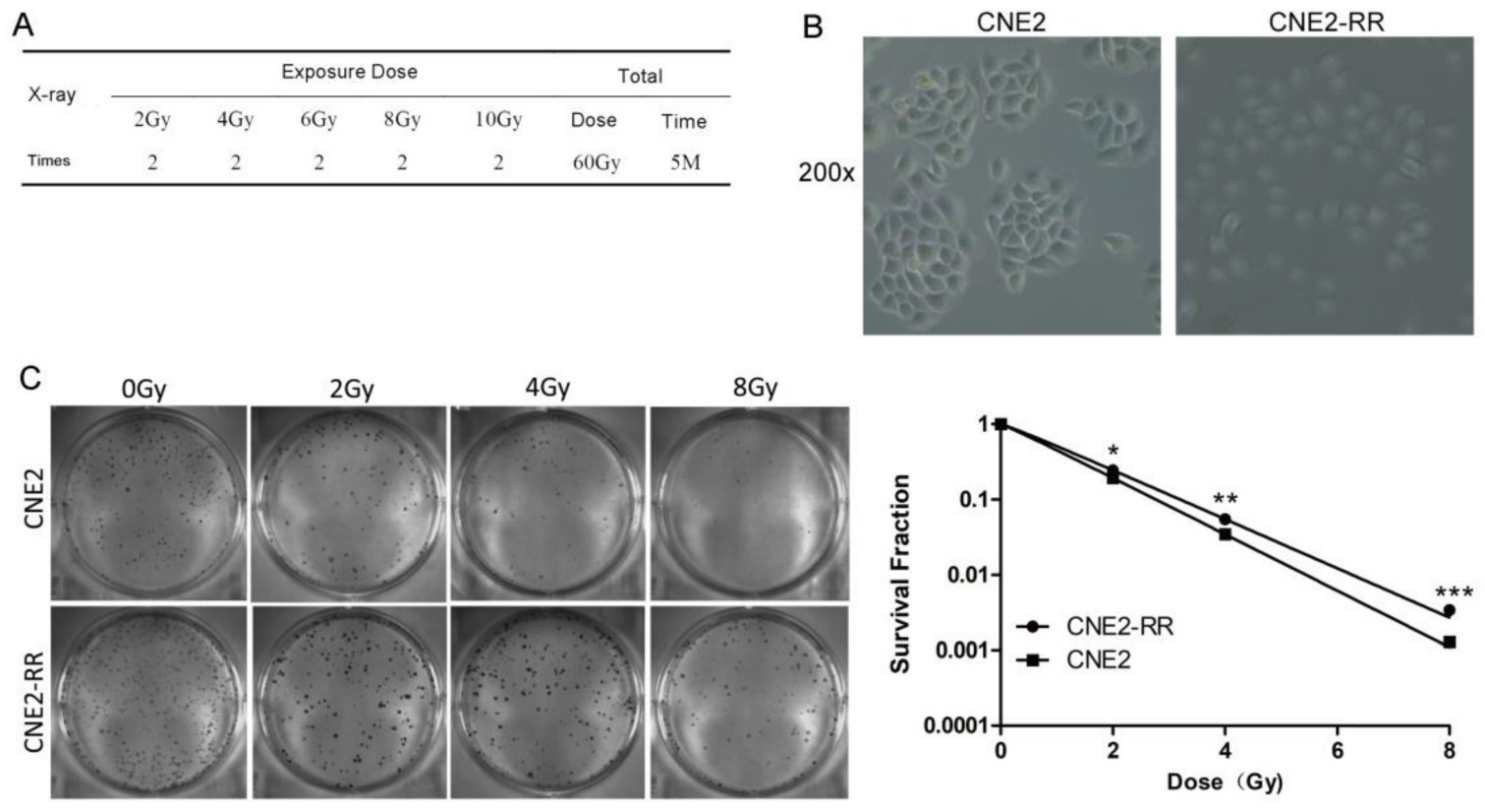
Establishment and validation of radioresistant NPC cell line. (A) The radiation scheme of NPC cell line CNE2. (B) The morphology of radioresistant CNE2-RR cell lines. (C) Typical images of colony formation and survival fraction for the different treatments are shown. *P<0.05, **P<0.01 and ***P<0.001.

**Figure 2:**
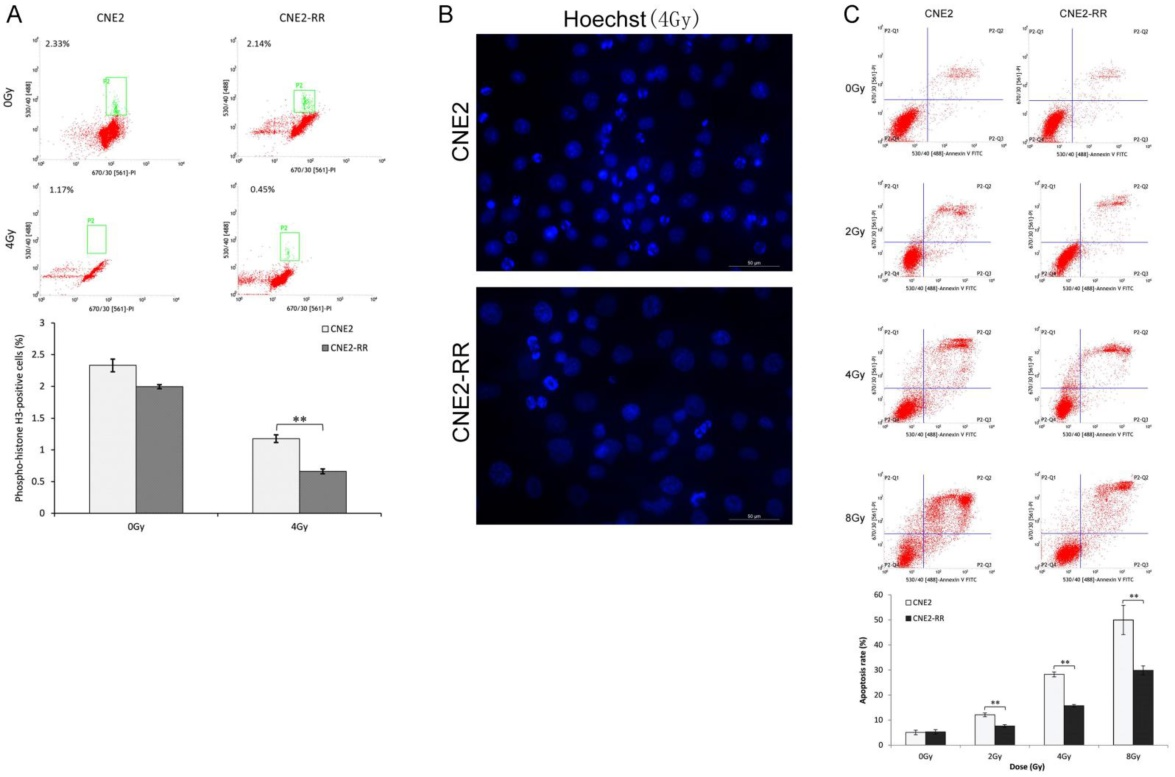
CNE2-RR can resist apoptosis. (A) Cells were untreated or irradiated as indicated, and mitotic cells were determined by FCM. Cells were exposed to IR, the apoptosis was determined by hoechst 33342 staining (B) and FCM (C). **P<0.01 and ***P<0.001.

The CNE2-RR cells were acquired by gradually increased the radiation dose and treated with fractionated irradiation and assessed for their ability to form colonies (Fig. 1A-C). CNE2-RR exhibited a stronger radioresistance to radiation at dose from 2 to 8 Gy, in contrast to parental CNE2 cells. The G2/M checkpoint function was determined by monitoring histone H3 phosphorylation on serine 10 21, 22. Clear reduction in phospho-H3-positive cells was observed in the CNE2-RR group cells post IR exposure, whereas a significant number of the CNE2 cells entered mitosis, indicating that the CNE2-RR cells have a strong ability to arrest the cell cycle in G2 phase compared with CNE2 cells (Fig. 2A). Considering the notion that cells can undergo apoptosis when DNA damage is irreparable, therefore, to examine whether CNE2-RR following IR treatment can resist apoptosis, cells were exposed to IR, and stained by Hoechst 33342, the results showed that the CNE2-RR group had fewer fragmented nuclei and nuclear shrinkage (Fig. 2B). In addition, we determined the apoptosis rate using FCM. IR potently induced apoptosis in CNE2 and CNE2-RR cells in a dose-dependent manner, however, CNE2-RR cells showed significantly more resistance to apoptosis compared with CNE2 (Fig. 2C). These results indicate that CNE2-RR cells were successfully established.

### DNA damage repair of CNE2-RR cells

**Figure 3:**
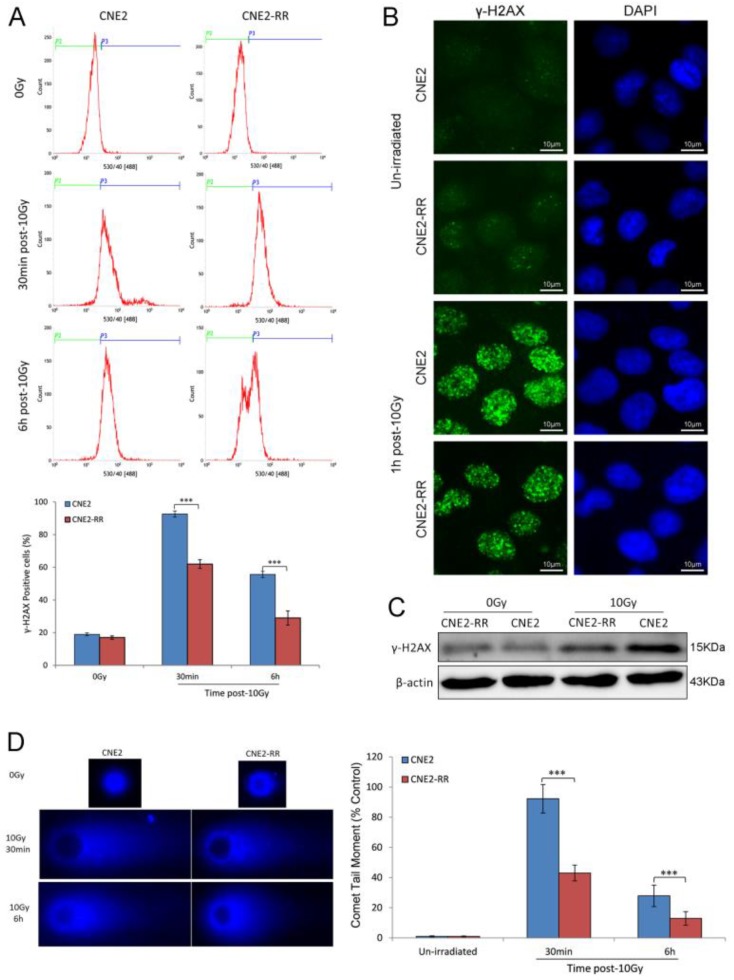
DNA damage repair of CNE2-RR. (A) Cells were untreated or irradiated as indicated, the mean percentage of γ-H2AX-positive cells was determined using FCM. (B) Representative γ-H2AX foci in CNE2-RR and CNE2 cells. Cells were untreated or irradiated with 10 Gy, fixed, stained with anti-γ-H2AX antibodies at the times indicated and detected by immunofluorescence. (C) Representative immunoblots of γ-H2AX expression are shown. (D) The ability of DNA damage repair was detected by comet assay. Representative images were on the left. The comet tail moment of 75 cells for each time and condition was quantified by CASP software and normalized to that of no irradiation. ***P<0.001.

DSBs are the most dangerous damage caused by IR, posing a serious threat to cell viability and genome stability. The expression of γ-H2AX, an indirect marker of DNA double-strand breaks (DSBs), was detected by FCM and western blotting. FCM analysis indicated that there were higher mean fluorescence intensity (MFI) and more γ-H2AX-positive cells in the CNE2 group than CNE2-RR group after IR treatment (Fig. 3A). Meanwhile, the γ-H2AX protein expression was significantly increased in CNE2 cells by western blotting (Fig. 3C). To confirm the inter-group difference of residual DNA damage, we further detected nuclear foci formation of γ-H2AX using immunofluorescence staining. The results showed IR-inducing γ-H2AX foci formation in CNE2-RR cells was less than CNE2 cells (Fig. 3B). Furthermore, we measured the persistence of DSBs utilizing single-cell gel electrophoresis (comet assay). The CNE2 group had more amounts of DNA damage compared to the CNE2-RR group at 30min and 6h after IR treatment (Fig. 3D). Therefore, the CNE2-RR cells have more resistance to radiation than parental CNE2 cells.

### Important signaling pathways associated with NPC radioresistance

**Figure 4:**
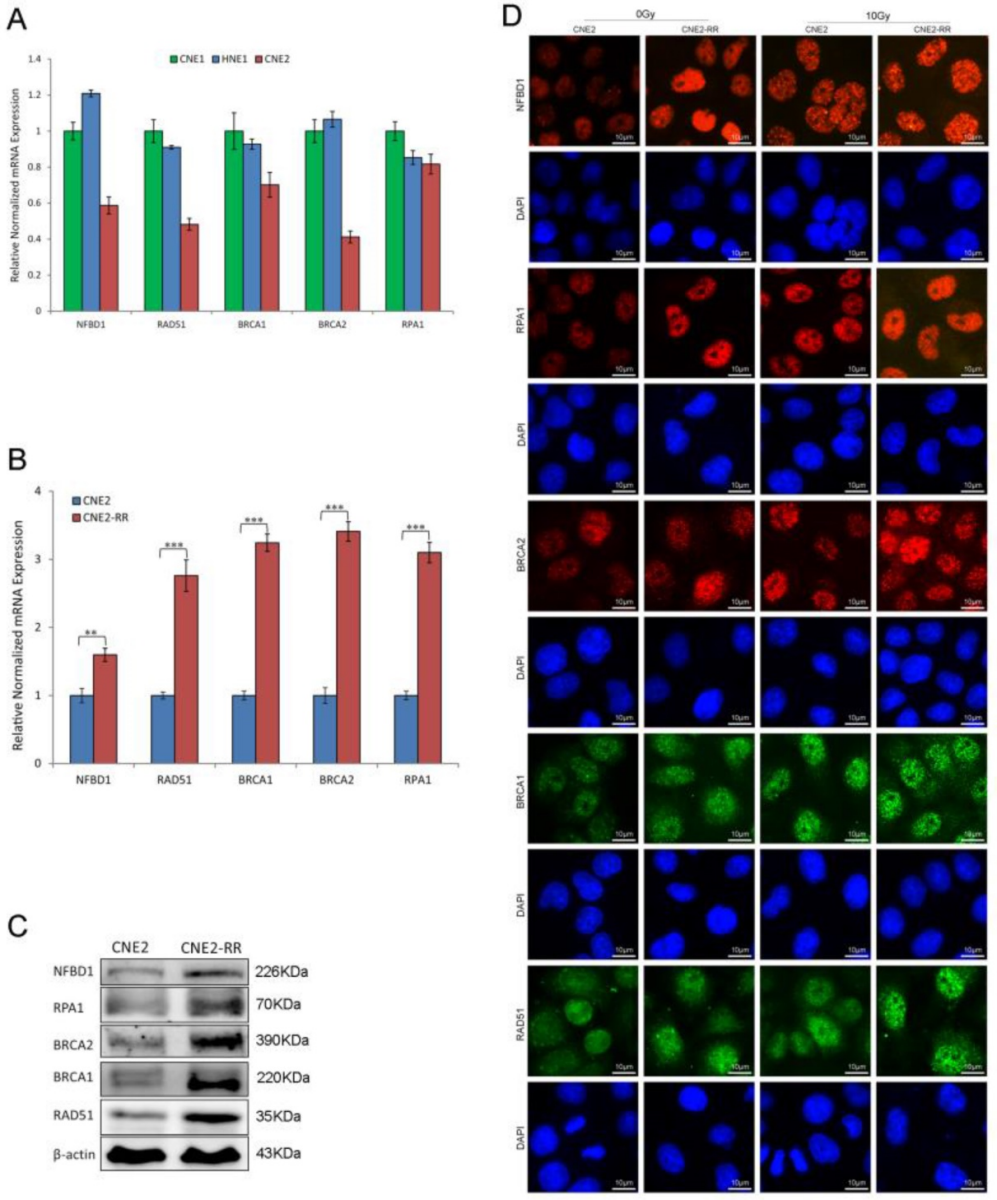
The HRR signaling pathway associated with NPC radioresistance. (A) The mRNA expression of HRR related genes (NFBD1, BRCA1, BRCA2, RPA1 and RAD51) was detected in CNE1, CNE2 and HNE1 by qRT-PCR. (B and C) The mRNA and proteins expression of HRR related genes were significantly increased in CNE2-RR compared with CNE2 cells. (D) IR significntly induced the HRR related proteins foci formation in CNE2-RR group compared to CNE2 group.

As IR cytotoxicity is mainly due to DNA damage, we explored the correlation between DNA damage repair genes and radioresistance, and found that the mRNA expression of NFBD1, BRCA1, BRCA2, RPA1 and RAD51 was significantly decreased in poorly differentiated NPC cell line CNE2 compared with well differentiated NPC cell lines CNE1 and HNE1 (Fig. 4A). Previous studies showed that the five genes are involved in the HRR pathway which has an important role in IR-inducing DNA damage response 14, 17, 23-26. Therefore, the HRR pathway maybe participate in the regulation of radioresistance in NPC. The mRNA expression of the genes in CNE2-RR group was increased compared with CNE2 group by qRT-PCR (Fig. 4B). Meanwhile, the five proteins expression was also significantly increased in CNE2-RR group by western blotting (Fig. 4C). Furthermore, immunofluorescence revealed corresponding increase in the protein levels, and IR significantly induced more foci formation of the proteins in CNE2-RR group compared to CNE2 group (Fig. 4D).

**Figure 5:**
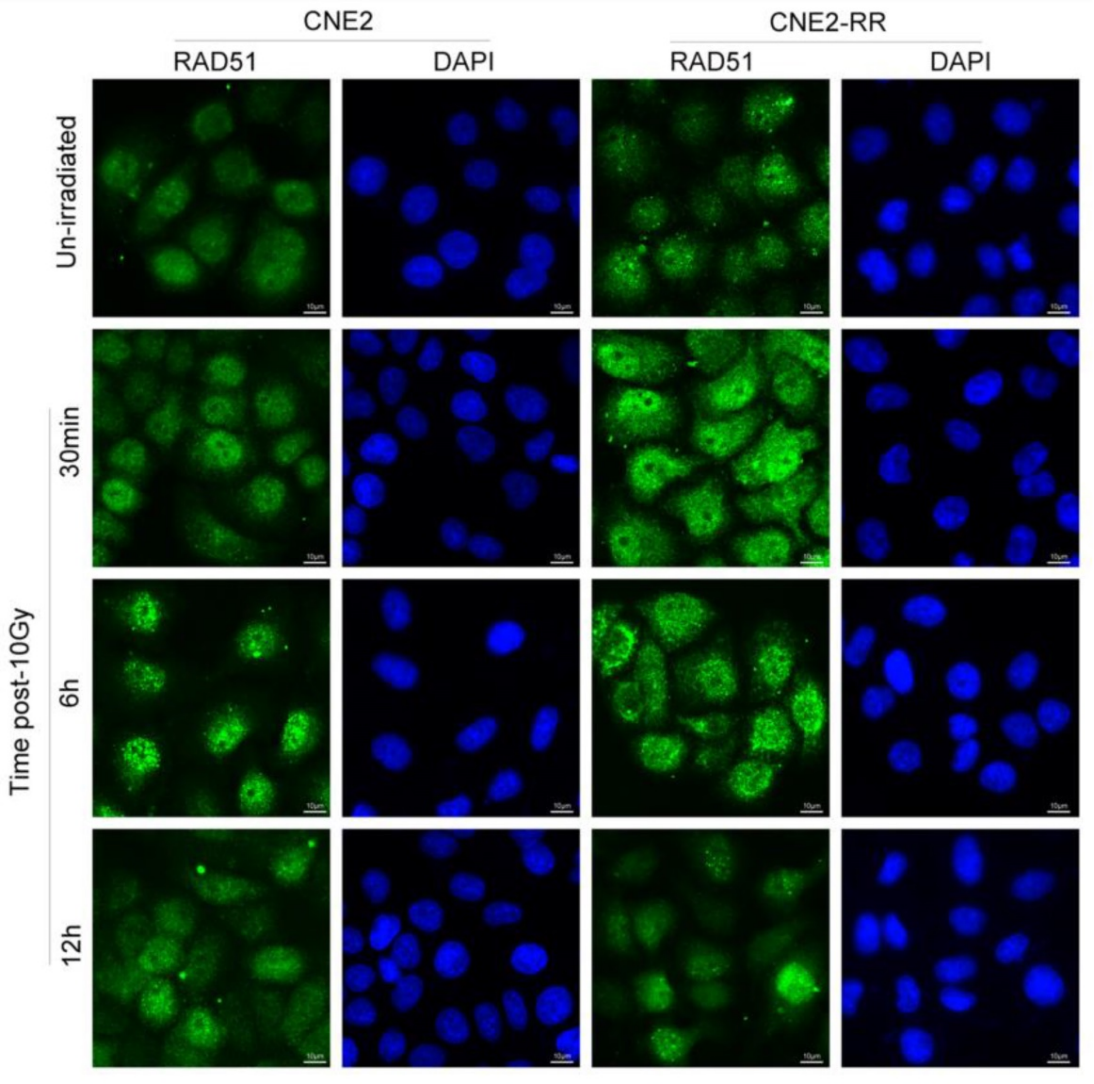
Representative RAD51 foci in CNE2-RR and CNE2 cells. The RAD51 foci-positive cells in CNE2-RR group contained both more and brighter foci than the CNE2 cells, especially at early time points. Cells were exposed to 10 Gy and fixed for immunofluorescence of RAD51 at the indicated times.

As previously reported, following IR treatment, the RAD51, which was respectively known as the representative protein of HRR pathway, was recruited to the DNA damage sites and formed subnuclear complex that were microscopically detectable as foci, which contained many of the enzymatic activities required for efficient repair of DSBs^23^. To further identify the relationship of HR with NPC radioresistance, we studied the foci formation of RAD51 at various time after IR treatment (Fig. 5). The formation of RAD51 foci at early time points (within 30min) was significantly different between CNE2-RR and CNE2 groups. In addition, the RAD51 foci-positive cells in CNE2-RR group contained both more and brighter foci than the CNE2 cells, especially at early time points. These results demonstrated that the HRR could be associated with radioresistance in NPC.

### Validation of the HRR pathway proteins in animal xenografts and NPC patients

**Figure 6:**
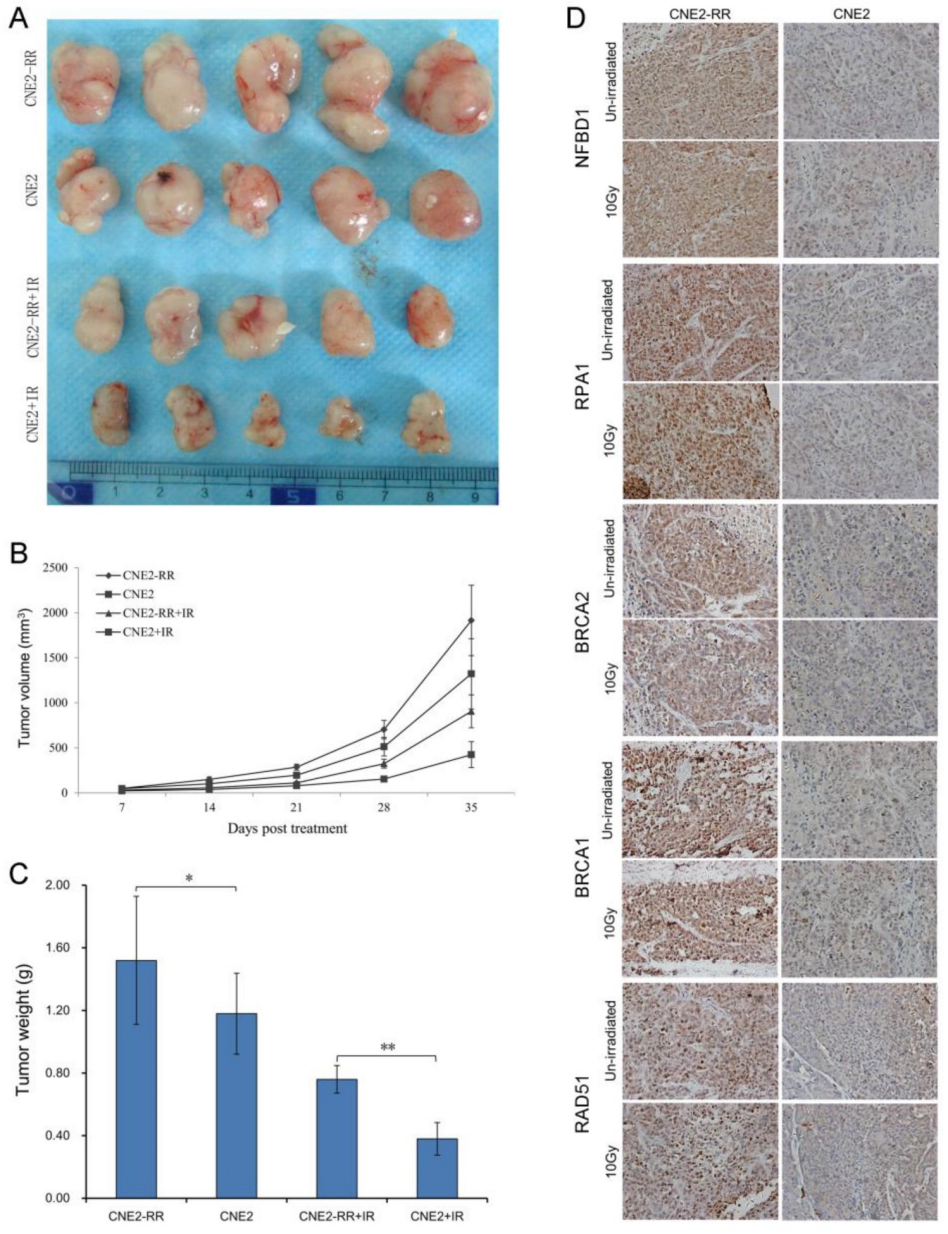
Validation of key pathway proteins from the HRR pathway in xenograft tumors. (A) Representative images of the tumors from mice 35 days after treatments. (B) The growth curves of CNE2 and CNE2-RR cells xenografts with radiation or non-radiation treatment. (C) Tumor weight at 35 days after treatments, n = 5 mice per condition. *P<0.05, **P<0.01. (D) The representative results of NFBD1, BRCA1, BRCA2, RPA1 and RAD51 in CNE2 and CNE2-RR cells xenograft tumors with radiation or non-radiation treatment by immunohistochemistry. *P<0.05 and **P<0.01.

To confirm the relationship between the main proteins from HRR pathway identified and NPC radioresistance in vivo, we next set up xenograft model in nude mice, and measured tumor volume once per week. Our results showed that either CNE2-RR or un-irradiation resulted in significantly larger tumor than CNE2 or irradiation lonely. Meanwhile, the CNE2 group resulted in significantly smaller tumors compared with the CNE2-RR group in the same IR treatment (Fig. 6A-C). Then the expressions of representative proteins including NFBD1, BRCA1, BRCA2, RPA1 and RAD51 proteins from the HRR pathway was found to be significantly increased in CNE2-RR animal xenografs by IHC (Fig. 6D). Furthermore, the cohort of 100 NPC patients consisted of 50 subjects with radioresistance and 50 subjects with radiosensitivity were recruited, and the demographic, clinical and pathologic characteristics of both groups are illustrated in supplementary Table S1.

**Figure 7:**
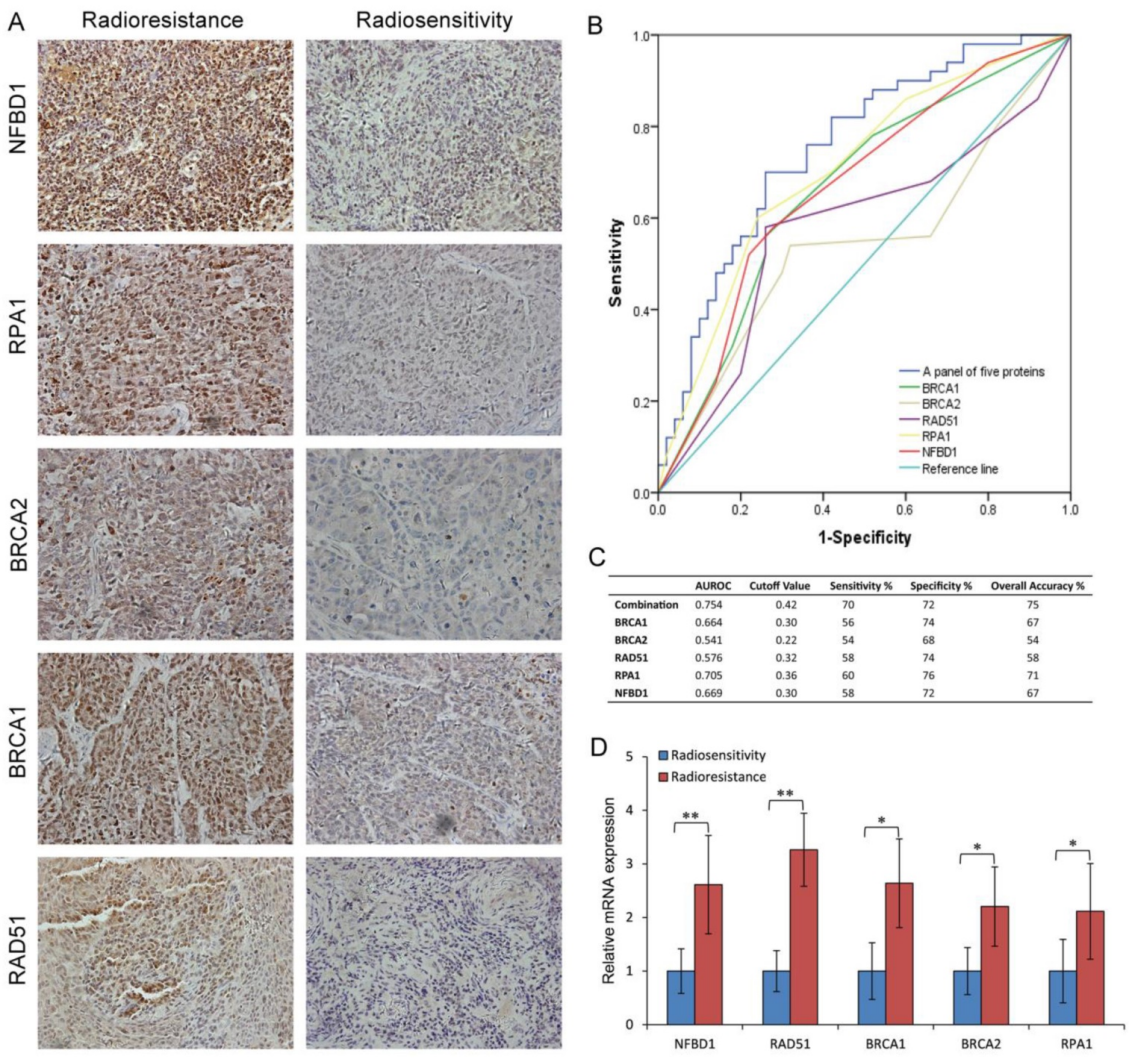
Expression of key pathway proteins from the HRR pathway in the radioresistant and radiosensitive NPC tissues. (A) The representative results of NFBD1, BRCA1, BRCA2, RPA1 and RAD51 in radioresistant and radiosensitive NPC tissues. (B) ROC curves for NFBD1, BRCA1, BRCA2, RPA1 and RAD51. (C) Youden Index cutoff values that maximized the sum of sensitivity and specificity were determined for each biomarker (crossed square on curve). The table provides performance values for each biomarker and as a panel. (D) The mRNA expression of NFBD1, BRCA1, BRCA2, RPA1 and RAD51 in radioresistant and radiosensitive NPC tissues.

To examine the link of these proteins to clinical NPC radioresistance, we detected the five-protein expression in the radioresistant and radiosensitive NPC tissues. As shown in Fig. 7A and supplementary Table S2, compared with the radiosensitive NPC, the expression of NFBD1, BRCA1, BRCA2, RPA1 and RAD51 proteins were upregulated in the radioresistant NPC. The same results were also found in the NPC tissue by qRT-PCR (Fig. 7D). The ability of the above biomarkers to predict the presence of NPC radioresistance was analyzed using nonparametric ROC analyses, according to National Cancer Institute guidelines 27. Based on the area under the ROC curve (AUROC) we determined Youden Index cutoff values to maximize the sum of sensitivity and specificity. The area under curve values of the five proteins and their sensitivity and specificity are listed in Fig. 7B and C together with their individual and collective values of merit. As a panel, the five proteins achieved a sensitivity of 70% and a specificity of 72% in discriminating radiosensitive from radioresistant NPC samples. These fndings indicate the HRR signaling pathways may play important roles in NPC radioresistance.

### Silencing NFBD1 enhances the radiosensitiity of CNE2-RR

**Figure 8:**
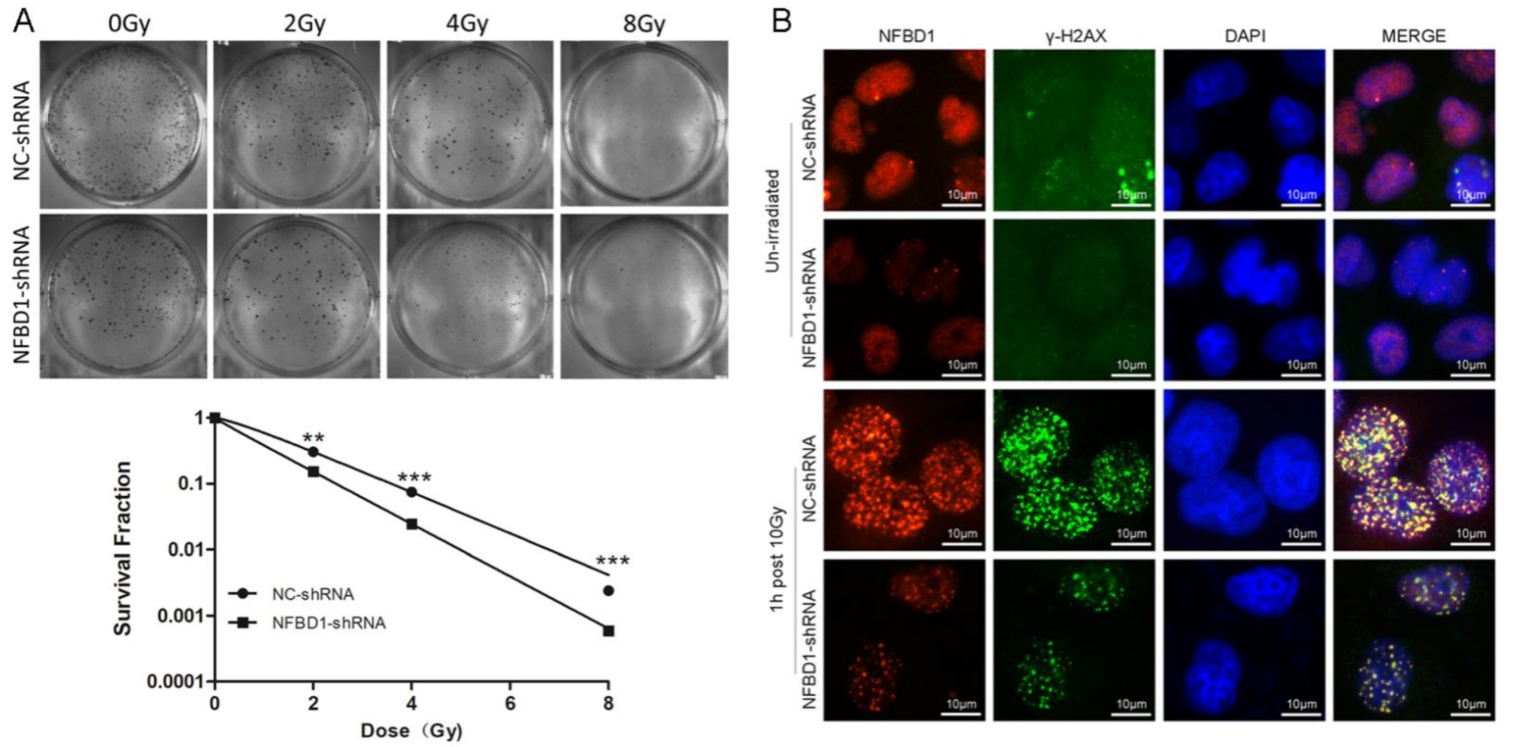
The effect of NFBD1 suppression on radiosensitivity of CNE2-RR cells. (A) Colony formation was significantly reduced in NFBD1-shRNA group compared with NC-shRNA group after IR treatment. (B) Silencing NFBD1 significantly inhibited the phosphorylation of H2AX after IR exposure, and the formation of γ-H2AX foci by immunofluorescence, and NFBD1 foci also significantly co-localized with γ-H2AX post irradiation. **P<0.01 and ***P<0.001.

To address the question whether the expression levels of the five proteins may affect the radiosensitivity of CNE2-RR, we selected NFBD1, one of the five proteins, to be further studied because our previous study showed that the downregulation of NFBD1 significantly enhanced the radiosensitivity and chemosensitivity through impairing IR-inducing the activity of HRR pathway in NPC cells 17, 18. As previously described 17, 18, we stably silenced the expression of NFBD1 in CNE2-RR cell line and measured the radioresistant levels of NFBD1-downregulation after irradiation with a range of radiation doses using the colony formation assay. As shown in Fig. 8A, silencing NFBD1 significantly increased the radiosensitivity. In response to DNA damage, many proteins involved in the DNA damage signaling pathway, including BRCA1, RPA1, and RAD51, are quickly recruited into IR-induced DSB sites in mammalian cells, and phosphorylation H2AX (γ-H2AX) may highlight the damaged chromatin 28. As NFBD1 controls the phosphorylation of several checkpoint-responsive proteins, we sought to examine whether it might have a role in H2AX phosphorylation. Silencing NFBD1 significantly impaired the phosphorylation of H2AX and the formation of γ-H2AX foci after IR exposure (Fig. 8B). Furthermore, NFBD1 foci also significantly co-localized with γ-H2AX post irradiation (Fig. 8B). In addition, IR induced a marked increase in RAD51 foci in shControl cells but not in shNFBD1 cells (Supplementary figure). The results indicate that NFBD1 confers a significant protection against ionizing radiation, and increased the resistance of NPC cells to irradiation.

## Discussion

In the current study, we successfully established radioresistant NPC cell line CNE2-RR by gradually increased the radiation dose. Furthermore, the radioresistance of CNE2-RR cells was evaluated by the colony formation, FCM and comet assays after a range of IR exposure. Our results demonstrated that CNE2-RR exhibited a stronger radioresistance to radiation, in contrast to parental CNE2 cells. It has been argued that cell cycle checkpoint arrest, although important for maintaining genomic stability post DNA damage, makes a less significant contribution to survival. However, when cell cycle checkpoint inactivation is combined with defective DSBs repair, the impact is more than additive, consistent with the notion that cell cycle checkpoint arrest enhances the opportunity for DSBs repair 29. In our studies, compared with control CNE2 cells, less CNE2-RR cells were entered in M phase after IR treatment, indicative of an active in G2/M checkpoint. Furthermore, the comet assay showed that CNE2-RR has stronger in radioresistance and DNA damage repair than parental CNE2 cells. From these data, the CNE2-RR cells are obviously radioresistant, which is suitable for studying the mechanism of NPC radioresistance.

To cope with DNA damage, cells possess a complex signaling network called the “DNA damage response”, which coordinates cell cycle control with DNA repair^30^-^32^. One especially important DNA repair pathway in this respect is the HR repair. The HRR pathway is an accurate form of DNA repair for a number of structurally distinct lesions and consequently essential for genome stability that involves multiple proteins and occurs during the S and G2 phase of the cell cycle 33. The broken DNA ends of a DSB are resected to allow invasion of the single strands into the sister chromatid, which functions as a template for accurate resynthesis of the damaged DNA. During HR-mediated repair of a DSB, the resulting broken ends are resected in a 5^`^- 3^`^ manner producing long stretches of 3^`^-single-strand DNA (ssDNA). Replication protein A (RPA) binds to the resulting ssDNA in order to avoid nuclease digestion and/or formation of secondary structures, and recruits the ATRIP-ATR complex, which signals to CHK1 to mediate S and G2 arrest. BRCA1 is phosphorylated by both ATM and ATR and consequently ubiquitinates CtIP to support the G2 checkpoint. BRCA2 delivers RAD51 displacing RPA and multimerization of RAD51 on ssDNA leads to the formation of a nucleoprotein filament for invasion and searching for complementary sequences on the sister chromatid to achieve error-free repair^34^-^36^. In the current study, we found that the five differential expression genes (NFBD1, BRCA1, BRCA2, RPA1 and RAD51) were involved in NPC radioresistance, which was further validated in CNE2-RR cells, tumor xenografs and NPC patients. In addition, using the Youden Index cutoff value, a panel of the five proteins analyses revealed a sensitivity of 70%, specificity of 72%. The above results suggest that the HRR pathway is potential biomarker for predicting NPC response to radiotherapy, and could be used as therapeutic targets for NPC radiotherapy.

NFBD1 (also known as KIAA01770 or MDC1) is an identified nuclear protein that regulates many aspects of the DNA damage-response pathway, such as intra-S phase checkpoint, G2/M checkpoint, and spindle assembly checkpoint 17, 37-40. Following DNA damage, NFBD1 serves as a bridging molecule and directly interacts with ATM and phospho-H2AX γ-H2AX) through its FHA and BRCT domains, respectively, which leads to the expansion of γ-H2AX region surrounding DNA strand breaks and provides docking sites for many DNA damage and repair proteins including the MRN complex, 53BP1, BRCA1 and so on, ensuring genomics stability 17, 18, 41-43. Our previous study showed that silencing NFBD1 impaired the HRR pathway, and enhanced the chemosensitivity and radiosensitivity in NPC cells 17, 18, 20, which indicates that NFBD1 upregulation may cause radioresistance in NPC. Our results showed that the upregulation of NFBD1 expresison was observed in the radioresistant NPC cells and tissues, and also associated with NPC radioresistance, meanwhile, silencing NFBD1 significantly enhanced the radiosensitivity of CNE2-RR cells. Furthermore, NFBD1 foci significantly co-localized with γ-H2AX foci, and depletion of NFBD1 impaired the IR inducing γ-H2AX foci formation. In addition, silencing NFBD1 could inhibit IR-induced the formation of RAD51 foci in nuclei. These data suggest that NFBD1 might be a therapeutic target for enhancing NPC response to radiotherapy.

In summary, our findings indicate that the HRR signaling pathway could have been involved in the NPC radioresistance, and controlling the HRR signaling pathway in combination with radiotherapy may hold promise to overcome NPC radioresistance.

## Supplementary Material

Supplementary figures and tables.

## Abbreviations

NPC: nasopharyngeal carcinoma
HR: homologous recombination
HRR: homologous recombineation repair
IR: ionizing radiation
NHEJ: non-homologous end-joining
IHC: immunohistochemistry
ssDNA: single-strand DNA
RPA: Replication protein A
DSBs: DNA double-strand breaks
qRT-PCR: real-time quantitative RT-PCR
DDR: DNA damage response
FCM: flow cytometry
