# Supplementary material for: The Homologous Recombination Repair Pathway is Associated with Resistance to Radiotherapy in Nasopharyngeal Carcinoma
Source: Int J Biol Sci. 2020 Jan 1;16(3):408–19. doi: 10.7150/ijbs.37302 (PMC6990897; doi:10.7150/ijbs.37302)
Supplement: Supplementary file 1 — Supplementary figures and tables. [file ijbsv16p0408s1.pdf]

Table S1. Clinicopathological Characteristics of radiosensitivity and radioresistance with NPC

Variable	Radiosensitivity	Radioresistance	Total	*P*
Age				
⩾45	34	37	71	
<45	16	13	29	0.660
Sex				
Male	36	30	66	
Female	14	20	34	0.291
Smoking				
+	26	30	56	
–	24	20	44	0.546
Drinking				
+	29	31	60	
–	21	19	40	0.838
T stage				
T1-T2	25	23	48	
T3-T4	25	27	42	0.841
Lymph node metastasis				
+	38	36	74	
–	12	14	26	0.820
TNM clinical stage				
I-II	20	15	35	
III-IV	30	35	65	0.402

Table S2. The expression of key pathway proteins in the radioresistant and radiosensitive NPC tissues

	n	Low expression	High expression	χ^2	*P*
NFBD1				9.653	0.002
Radioresistance	50	11	39		
Radiosensitivity	50	26	24		
RPA1				13.300	0.000
Radioresistance	50	12	38		
Radiosensitivity	50	30	20		
BRCA2				4.937	0.026
Radioresistance	50	16	34		
Radiosensitivity	50	27	23		
BRCA1				9.301	0.002
Radioresistance	50	13	37		
Radiosensitivity	50	28	22		
RAD51				9.180	0.002
Radioresistance	50	14	36		
Radiosensitivity	50	29	21		

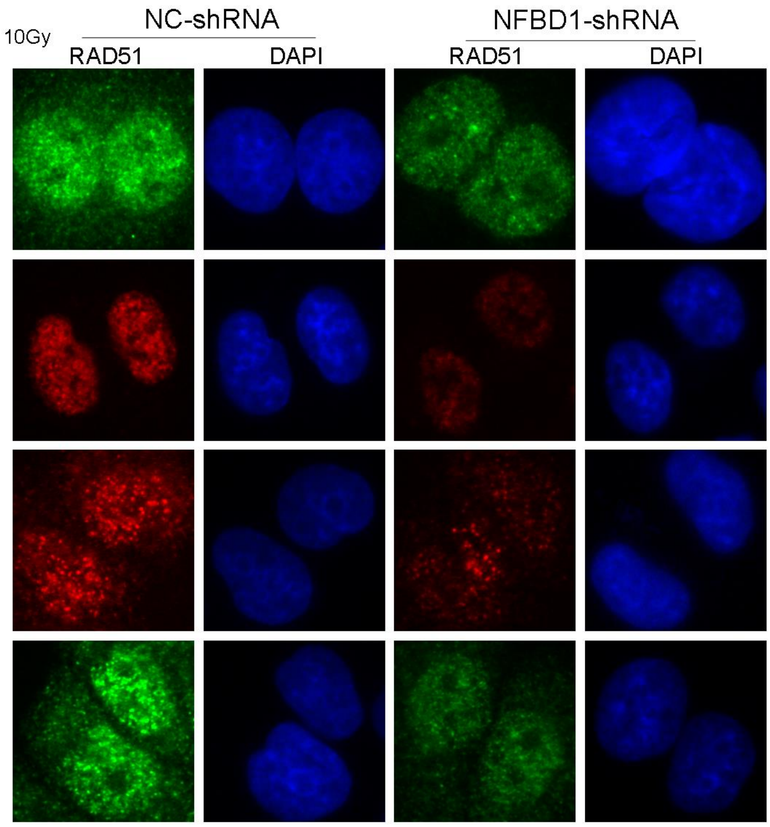

Supplementary Figure Silencing NFBD1 inhibits IR-induced the formation of the HR proteins foci in nuclei.
